# mTORC1 Activation by Loss of *Tsc1* in Myelinating Glia Causes Downregulation of Quaking and Neurofascin 155 Leading to Paranodal Domain Disorganization

**DOI:** 10.3389/fncel.2018.00201

**Published:** 2018-07-12

**Authors:** Qian Shi, Julia Saifetiarova, Anna Marie Taylor, Manzoor A. Bhat

**Affiliations:** Department of Cellular and Integrative Physiology, School of Medicine, University of Texas Health Science Center at San Antonio, San Antonio, TX, United States

**Keywords:** tuberous sclerosis complex, mTORC1, myelination, quaking, neurofascin, node of ranvier, paranodal organization

## Abstract

Mutations in human tuberous sclerosis complex (TSC) genes *TSC1* and *TSC2* are the leading causes of developmental brain abnormalities and large tumors in other tissues. Murine *Tsc1/2* have been shown to negatively regulate the mammalian target of rapamycin complex 1 (mTORC1) signaling pathway in most tissues, and this pathway has been shown to be essential for proper oligodendrocytes/Schwann cell differentiation and myelination. Here, we report that ablation of *Tsc1* gene specifically in oligodendrocytes/Schwann cells activates mTORC1 signaling resulting in severe motor disabilities, weight loss, and early postnatal death. The mutant mice of either sex showed reduced myelination, disrupted paranodal domains in myelinated axons, and disorganized unmyelinated Remak bundles. mRNA and protein expression analyses revealed strong reduction in the RNA–binding protein Quaking (Qk) and the 155 kDa glial Neurofascin (Nfasc^NF155^). Re-introduction of exogenous *Qk* gene in *Tsc1* mutant oligodendrocytes restored Nfasc^NF155^ protein levels indicating that Qk is required for the stabilization of Nfasc^NF155^ mRNA. Interestingly, injection of Rapamycin, a pharmacological mTORC1 inhibitor, to pregnant mothers increased the lifespan of the mutant offspring, restored myelination as well as the levels of Qk and Nfasc^NF155^, and consequently the organization of the paranodal domains. Together our studies show a critical role of mTORC1 signaling in the differentiation of myelinating glial cells and proper organization of axonal domains and provide insights into TSC-associated myelinated axon abnormalities.

## Introduction

The mammalian target of Rapamycin (mTOR) is a central player in mTOR signaling pathway, governing the cellular growth and metabolic homeostasis in response to many growth factors, cellular energy status and amino-acid levels (Saxton and Sabatini, 2017a). mTORC1 is activated by Rheb, which is inhibited by a GTPase-activating heterodimeric protein complex, formed by tuberous sclerosis complex (TSC) proteins, Hamartin (TSC1) and Tuberin (TSC2). Mutations in human *TSC1* or *TSC2* have been associated with a multisystem disorder in an autosomal-dominant manner. Disruption of TSC complex results in increased phosphorylation of mTORC1 and its downstream targets, including ribosomal protein S6 kinase (S6K) and 4EBP1, with subsequent elevation in protein translation. Due to the broad spectrum of tissue distribution of mTORC1 signaling, TSC affects many important organs, such as the brain, kidney and lungs (Crino et al., 2006).

TSC severely affects neuronal function, both in CNS and PNS, leading to cognitive impairment, epilepsy, autism and white matter abnormalities (Ruppe et al., 2014; Ercan et al., 2017). Severe hypomyelination and loss of OLs are the hallmarks of white matter defects observed in the CNS (Lewis et al., 2013; Peters et al., 2013). While accumulated evidence has shown that mTORC1 is the essential regulator of development and oligodendrocyte differentiation (Lebrun-Julien et al., 2014), the exact function of mTORC1 is still being investigated. Given the role of mTORC1 signaling in regulating protein translation, it is surprising to find that activation of mTORC1 by *Tsc1* or *Tsc2* deletion in oligodendrocyte progenitor cells (OPCs) leads to hypomyelination, which has been recently reported (Lebrun-Julien et al., 2014; Carson et al., 2015; Jiang et al., 2016). It appears that a delicate balance of mTOR signaling is required for the differentiation of oligodendrocytes, as either hyper-activation or inactivation of mTORC1 signaling leads to hypomyelination and disruption of OPC differentiation. The temporal activation of mTORC1 signaling also plays a critical role in determining the fate of OPCs. A recent study showed that *Tsc1* depletion from OL progenitor cells accelerated remyelination; however, *Tsc1* deletion from differentiated OLs slowed remyelination, again suggesting a rather complex role of mTOR signaling in determining OL differentiation and myelination (McLane et al., 2017).

Despite the wide spectrum of studies on mTORC1 signaling, its role in the process of myelination and associated CNS/PNS disorders remains to be fully understood. In the current study, we sought to elucidate the role of elevated mTORC1 signaling in CNS/PNS myelination by generating *Tsc1* mutant animals specifically in myelinating OLs and SCs. Ablation of *Tsc1* resulted in severe motor disabilities, weight loss, and a short postnatal lifespan. Most importantly, *Tsc1* deficiency in myelinating glia leads to severe hypomyelination and disorganization of PNS Remak bundles. We also show that loss of *Tsc1* results in reduced *Qk* and *Nfasc*^NF155^ mRNA and protein levels associated with either complete absence of the paranodes or asymmetric presence of paranodes. Re-expression of *Qk* in *Tsc1* mutant OPCs restored *Nfasc*^NF155^ mRNA and protein levels, indicating that Qk is essential for *Nfasc*^NF155^ mRNA stability and Nfasc^NF155^ protein levels in myelinating glial cells. Interestingly, the phenotypic abnormalities observed in *Tsc1* mutants were significantly rescued by injection of Rapamycin at early developmental stages. However, the Rapamycin administered mice eventually developed severe motor disability and died around postnatal day (P) 25, indicating that continuous activation of mTORC1 pathway is detrimental to normal neural development. Together our data provide novel evidence into mTORC1 signaling and axonal domain organization through Qk and Nfasc^NF155^ and suggest that Rapamycin-dependent pharmacological intervention may be helpful in managing some of the myelin-related abnormalities in TSC-associated disorders.

## Materials and Methods

### Generation of Tsc1 ^Flox/Flox^;Cnp-Cre (Tsc1^cKO^) Mice

All animal experiments were performed according to the University of Texas Health Science Center at San Antonio-Institutional Animal Care and Use Committee approved guidelines for ethical treatment of laboratory animals. *Tsc1^Flox^* (#005680) mouse strain was purchased from Jackson Laboratory. *Cnp-Cre* mouse was kindly provided by the Nave laboratory (Lappe-Siefke et al., 2003). All animal research was performed with prior approval from UT Health San Antonio’s Institutional Animal Care and Use Committee and conforms to the Public Health Service Policy on Humane Care and Use of Laboratory Animals.

### Antibodies

Anti-βIV Spectrin, anti-Caspr, pan anti-Neurofascin (NFCT), anti-NF155 were described previously (Pillai et al., 2009; Thaxton et al., 2011; Saifetiarova et al., 2017b; Taylor et al., 2017). Other primary antibodies used in this study were rabbit anti-Tsc1 (#6935, Cell Signaling), rabbit anti-MBP (ab40390, Abcam), mouse anti-Caspr (75-001, NeuroMab), mouse anti-Kv1.2 (75-008, NeuroMab), mouse anti-β-Actin (A-5441, Sigma), anti-human tuberin (#3635s, Cell Signaling), anti-S6 kinase (#2708s, Cell Signaling), anti-phospho-S6 kinase (T389; #9205s), anti-pan-Qk (N147/6, NeuroMab), anti-PDGFRa (#558774, BD Biosciences), anti-PMP22 (PA567643, Invitrogen), anti-MZP (P0; ABN363MI, EMD Millipore) and anti-MAG (a generous gift from Dr. James L. Salzer). Fluorophore-conjugated secondary antibodies used for immunofluorescence and HRP-conjugated secondary antibodies used for immunoblotting were purchased from Invitrogen. Infrared (IR) conjugated secondary antibodies used for immunoblotting were purchased from LI-COR.

### Tissue Preparation and Immunostaining

Animals were deeply anesthetized with 2% Avertin (T48402, Sigma) and transcardially perfused with PBS for 4–5 min, followed by ice-cold 4% paraformaldehyde (PFA) in PBS. Consistently, throughout our studies, 1 cm lateral tracts of the cervical spinal cord (SC) was dissected out and post-fixed in the same fixative overnight at 4°C and then immersed in 30% sucrose in PBS until it settled at the bottom. The tissue was rinsed three times extensively in PBS, frozen in Tissue-Tek O.C.T. Compound (Sakura Finetek USA, Inc., Torrance, CA, USA), and kept in −80°C freezer for further usage. Longitudinal 14 μm sections were cut with a cryostat (Leica, Germany), mounted on slides and immediately immunostained. Distal sciatic nerves (SN) were dissected out from anesthetized animals before perfusion, fixed in 4% PFA for 30 min, washed carefully in PBS and teased on slides to individual nerve fibers. Slides with teased SN were dried overnight at room temperature and stored at −80°C. Immunostaining of all samples was carried out as previously described (Saifetiarova et al., 2017b; Taylor et al., 2017). Slides were coverslipped with anti-fade mounting media. Immunostaining images were acquired by Zeiss LSM 710 confocal microscope with 40× magnification using identical settings. Optical fields were randomly selected throughout the slide and at least 10 independent images for each control and mutant animal were acquired. On average at least 100 nodes/paranodes for PNS and 200 nodes/paranodes for CNS from each animal were collected for quantification analysis.

### Immunoblotting

Cells or tissues were collected/homogenized and lysed on ice in RIPA buffer (25 mM Tris-HCl, pH 7.5, 150 mM NaCl, 1 mM EDTA, 1% NP-40 and 5% glycerol) with protease inhibitors (#88660SPCL, Thermo Fisher Scientific) and phosphatase inhibitor mix (sc-45044, Santa Cruz). The lysates were sonicated for 10 s, before centrifugation at 13,000 *g* at 4°C for 30 min. Total protein concentration was quantified by Pierce BCA protein assay kit (Thermo Scientific). The supernatant was collected as a final lysate with 6× sample buffer, heated for 5 min at 100°C boiling water. Equal amounts of protein were subjected to 10% or 4%–12% SDS-PAGE gels depending on the protein of interest. The proteins were then transferred to nitrocellulose membranes and probed with primary antibodies as previously described (Thaxton et al., 2011). Afterwards, membranes were incubated in IR-conjugated secondary antibodies followed by detection of the probes, and visualized under LI-COR. The intensities of western blot bands were quantified by ImageJ software (NIH), normalized to β-Actin or GAPDH and one of the control samples.

### Transmission Electron Microscopy

Animals were anesthetized and transcardially perfused with normal saline followed by 5% glutaraldehyde/4% PFA EM fixative for 30 mins. After perfusion, entire mouse carcasses were post-fixed for another 2 weeks in the same EM fixative at 4°C. SN and SC then were dissected out and incubated overnight in 0.1 M sodium cacodylate buffer followed by incubation in 2% OsO_4_ solution and gradient ethanol dehydration. Finally, samples were incubated in propylene oxide, left in 100% PolyBed resin with constant agitation for 36 h at room temperature and embedded in flat molds at 55°C for 36 h. After embedding, samples were submitted to the UTHSCSA Electron Microscopy Lab and processed as previously described (Green et al., 2013). Briefly, tissue was sliced in 90 nm sections and placed on copper grids. Grids were stained with uranyl acetate for 30 s in the microwave and then with Reynold’s lead for 20 s. Samples were imaged on a JEOL 1230 electron microscope using advanced microscopy techniques (AMT) software. For quantification of myelinated axons, at least 20 images were obtained at 5600× for each tissue, and three animals per genotype/experimental condition were prepared. At least 200 myelinated or unmyelinated axons were quantified in each animal. For Remak bundle quantification, 200 axons in Remak bundles and 10 Remak bundles per animal were quantified.

### *In Vivo* Nerve Conduction Measurements

The conduction velocity measurements of sciatic nerves were carried out on at least five separate *Tsc1^Flox^* and *Tsc1^cKO^* mice at P10 and P15 as described previously (Saifetiarova et al., 2017b; Taylor et al., 2017). Briefly, pups were anesthetized by continuous isoflurane (5% aerosolized) while electrophysiological recordings were collected from the sciatic nerve using a Nicolet Teca Synergy portable neurological system (Natus Neurology Inc., Middleton, WI, USA). Recording electrodes were placed in the dorsum of the foot, and two separate recordings were made: at the ankle (0.2 ms, 10 mA) and the sciatic notch (0.2 ms, 10 mA). To determine NCV, the distance between the notch and ankle divided by the difference between the notch and ankle latencies was calculated. For amplitude, the difference in mV from the onset to the peak of the compound action potential was determined. After the recordings, each pup was monitored as it recovered from anesthesia and then returned to its home cage.

### Isolation and Differentiation of Mouse OPCs and Transfection of DNA

Isolation of the mouse OPCs were performed as described by Chen et al. (2007a). Briefly, the forebrains from P5 to P7 pups were removed, diced into 1 mm fragments and incubated at 37°C for 15 min with trypsin and DNase. Dissociated cells were plated on poly-L-lysine-coated tissue culture flasks and grown at 37°C for 10 days in DMEM medium with 20% fetal calf serum (Invitrogen). OPCs were collected by shaking the flask overnight at 250 rpm at 37°C, resulting in 95% purity. Cultures were seeded into 12 well plates and maintained in high-glucose DMEM medium containing Forskolin (4.2 μg/mL) and CNTF (10 ng/mL). PDGF (10 ng/mL) and NT-3 (1 ng/ml) were added into the medium to promote OPC proliferation. After a week of culture, differentiation of OPCs was induced by addition of T3 at 30 ng/ml, and withdrawal of PDGF, for 3+ days. The differentiated oligodendrocytes were counted based on their characteristic morphology and the staining for myelin basic protein (MBP) and Cnpase. All growth factors were purchased from Peprotech, Rocky Hill, NJ, USA.

Wild-type *Qk* plasmid was purchased from Addgene (#19891). The control plasmid *pcDNA 3.1* or *FLAG-Qk* plasmid were transfected into OPCs (4.5 × 10^4^ cells per well in 12 well plate) using Lipofectamine 2000 (Invitrogen) for 48 h, and the cells were cultured in OPC-Differentiation medium with T3 for total 3 days.

### RNA Extraction and RT-PCR Analyses

Total RNA from cells or tissues was isolated by using PureLinkTM RNA mini kit (Ambion) with Trizol reagent (Invitrogen). cDNA reverse transcription was performed with High Capacity cDNA Reverse Transcription kit (Applied Bio System). Sequences of primers listed in Table 1 are designed spaning the exons and synthesized by Eurofins Genomics (Louisville, KY, USA). Quantitative RT-PCR was performed with Power SYBR PCR Master Mix (Applied Bio System) on ABI 7900HT Fast Real-Time PCR System. Data were normalized to β-actin and controls mRNA levels using 2^−ΔΔCt^ method (Livak and Schmittgen, 2001). One of the control samples served as a reference for quantification for both control and mutant samples.

**Table 1:** List of primers used for analysis of oligodendrocyte differentiation.

Gene	Genebank ID	Forward primers	Reverse primers
Pdgfra	NM_001083316.2	TCCATGCTAGACTCAGAAGTCA	TCCCGGTGGACACAATTTTTC
O4	NM_008770	CCACCTGCCGAAAAATGGAC	CACGTAGCCTGGAAGGATGAG
St8sia1	NM_011374.2	GAAGAAATGTGCGGTGGTGG	GGTTGCACCGCATGACAAA
Oligo2	NM_139001.2	ACACAGGCCTGCAAATCTGG	CTGGGCCCGAATCATTGTCT
Nkx2	NM_001077632	AAGCATTTCAAAACCGACGGA	CCTCAAATCCACAGATGACCAGA
Sox10	NM_011437	CAAGCTCTGGAGGTTGCTGA	CCGGATGGTCCTTTTTGTGC
Ugt8a	NM_011674	ACTCCATATTTCATGCTCCTGTG	AGGCCGATGCTAGTGTCTTGA
MBP	XM_021150283	ACCCCTGTCACCGCTAAAGA	TTCCTCCCAAGGCACAGAGA
GADPH	NM_008085	TACTGTTGTCCAGCTACGGC	GTGGTCTGATCACAGGGCAT

### g-ratio Measurements

The circumferences of fibers and axons were measured using ImageJ (NIH). The *g*-ratio was determined by (axon circumference)/(fiber circumference). The *g*-ratio was only calculated for myelinated axons. At least 20 images/animal taken at 5600× magnification were used for the *g*-ratio measurement. The *g*-ratio was measured for at least 50 axons in each animal (*n* = 3).

### Rapamycin Treatment

Rapamycin powder (Fisher Scientific) was dissolved in DMSO and stored at 10 mM in aliquots at −80°C. On the day of injection, working solution was prepared freshly with a final concentration of 1 mg/ml rapamycin in 0.25% Tween-20 and 0.25% PEG400 (PBS). Pregnant moms were administered daily intraperitoneal injections with either rapamycin (10 mg/kg body weight) or vehicle once per day from E19 to P3. Tissues from mice at P15 were then collected and processed for analyses.

### Statistics

All data are presented as mean ± SEM. Statistically significant differences between mutant and control groups were determined by Student’s *t*-tests for two groups, ordinary two-way ANOVA (none repeated measures) for two factor multiple groups, and post-test analysis was performed using Tukey test, using GraphPad Prism six software and are represented by *p < 0.05, **p < 0.01 or ***p < 0.001.

## Results

### Ablation of *Tsc1* in Myelinating Glia Results in Motor Disability and Decline in Peripheral Nerve Conduction Properties

**Figure 1:**
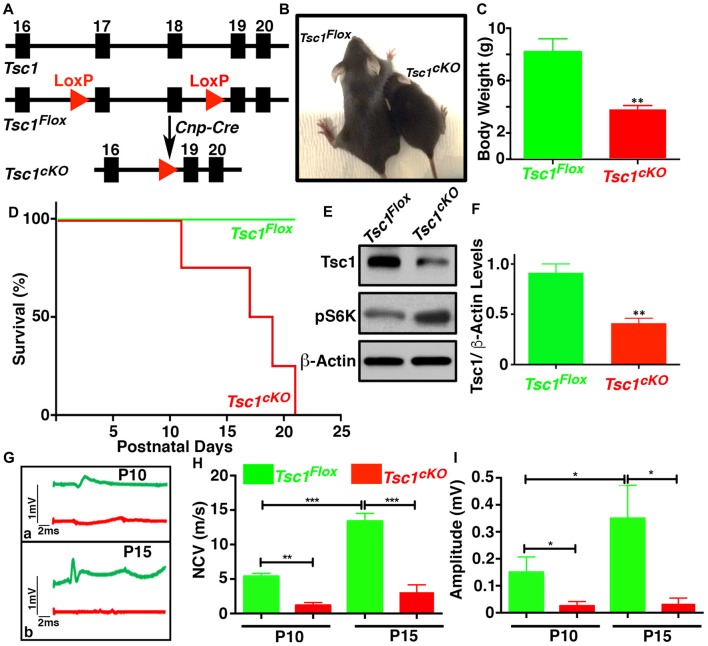
Ablation of *Tsc1* in myelinating glia results in motor disability and decline in peripheral nerve conduction properties. **(A)** Schematic diagram showing the partial genomic map of the *Tsc1* locus. *Tsc1* exons 17 and 18 are flanked by *LoxP* sites which allow Cre-mediated excision of the floxed axons. **(B)** Photographs of control (*Tsc1^Flox^*) and *Cnp-Cre;Tsc1^Flox^* *(Tsc1^cKO^*) mice at P10. **(C)** Body weight measurement of *Tsc1^Flox^* (green bar) and *Tsc1^cKO^* (red bar) mice at P10. *n* = 5, comparisons between genotypes were performed by *Student* *t*-test. **p < 0.01. **(D)** Survival curve of *Tsc1^Flox^* and *Tsc1^cKO^* mice. *n* = 5 in each group. **(E)** Expression of Tsc1 and pS6K in the spinal cords (SCs) of *Tsc1^Flox^* and *Tsc1^cKO^* mice at P15 examined by western blot analysis. β-actin was used as a loading control.* n* = 5. **(F)** Quantification of intensities of Tsc1 levels measured by ImageJ software (NIH). Comparisons between genotypes were performed with *Student* *t*-test. *n* = 5, **p < 0.01. **(G–I)** Representative electrophysiological profiles of CAPs from sciatic nerves are shown in **(G)**. Quantification of nerve conduction velocity (NCV) **(H)** and amplitude **(I)**, respectively, from sciatic nerves of P10 and P15, *Tsc1^Flox^* and *Tsc1^cKO^* mice. *n* = 5, comparisons between genotypes or between different ages within the same genotypes were performed by two-way ANOVA with Tukey Statistical post-test *p < 0.05, **p < 0.01 or ***p < 0.001.

Earlier studies on *TSC* mouse models have reported hypomyelination in the nervous system (Lebrun-Julien et al., 2014; Carson et al., 2015; Jiang et al., 2016). To better understand the role of mTORC1 signaling in myelinating glia during development, we generated *Tsc1* conditional knockout mice by breeding *Tsc1^Flox/Flox^*(designated *Tsc1^Flox^*) mice with the 2’,3’-cyclic nucleotide 3’-phosphodiesterase *(Cnp)-Cre* mice, which specifically cause the deletion of exon 17 and 18 in the*Tsc1* locus in OLs and SCs (Figure 1A). The *Tsc1^Flox^*;*Cnp-Cre* (designated *Tsc1^cKO^*) mice were born at the normal Mendelian ratio and showed neurological phenotypes, characterized by hind-limb paresis, rapid tremor, and ataxia, starting at around P10. The *Tsc1^cKO^* mice were significantly smaller in size (Figure 1B), and their body weights were only half compared to their *Tsc1^Flox^* control littermates at P10 (Figure 1C). The *Tsc1^cKO^* mice had a much shorter median lifespan (~15 days; Figure 1D). As expected, immunoblot analysis of the SC lysates from *Tsc1^cKO^* mice showed decreased levels of Tsc1 (Figures 1E,F) and accordingly increased levels of phosphorylated S6 ribosomal kinase (pS6K, Figure 1E) suggesting the activation of mTORC1. Since the *Tsc1^cKO^* mutant mice showed severe motor function deficits, we analyzed the nerve conduction properties by performing *in vivo* recording of the sciatic nerves of *Tsc1^cKO^* mutants and control littermates at both P10 and P15. Starting at P10, the nerve conduction velocity (NCV) values (Figures 1Ga,H) and amplitudes at the notch (Figures 1Gb,I) were significantly reduced in the sciatic nerves of *Tsc1^cKO^* mutants (see comparisons in Figures 1H,I). Although the NCV and amplitudes increased with age in control littermates, in the *Tsc1^cKO^* mutants, this increase was not significant indicating a severe impairment in nerve conduction properties. Taken together, these data show that Tsc1 function in myelinating glia is critical for proper neural development and nerve function.

### Loss of Tsc1 in Myelinating Glia Affects the Myelination Phase Leading to Axonal Hypomyelination

**Figure 2:**
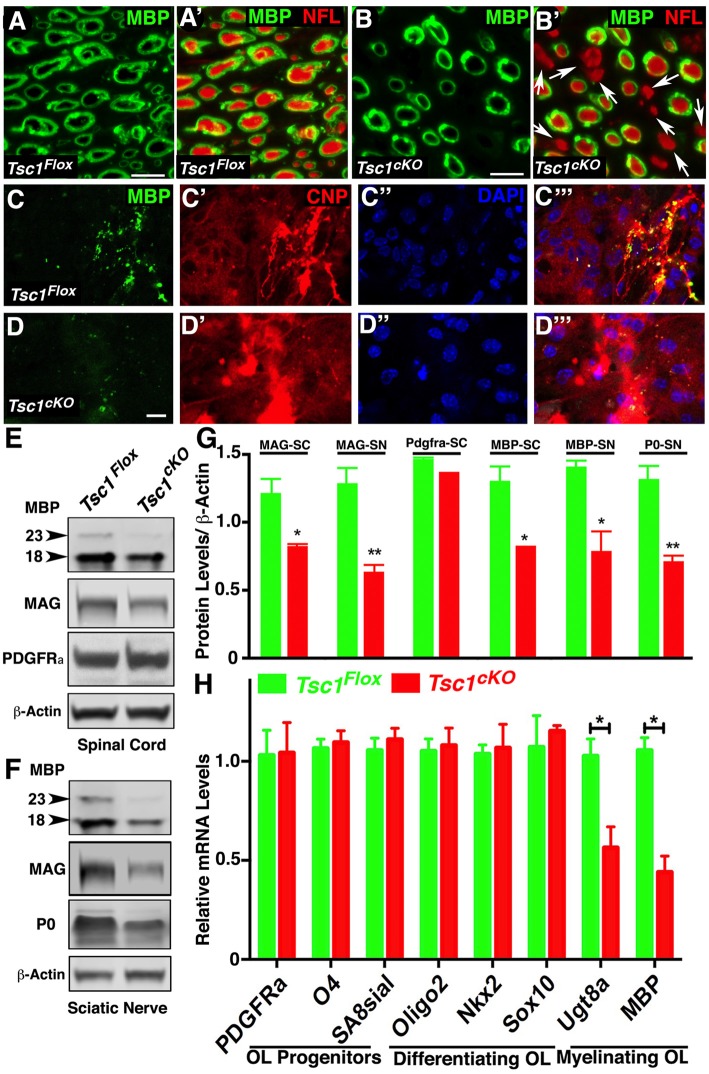
Loss of Tsc1 in myelinating glia affects the myelination phase leading to axonal hypomyelination. **(A,B)** Immunostaining of cross cryosections of sciatic nerves from P15 *Tsc1^Flox^* **(A)** and *Tsc1^cKO^* **(B)** mice with antibodies against myelin basic protein (MBP; green; **A,A’,B,B’**) and pan-Neurofilament (NFL, red, **A’,B’**). White arrows indicate axons lacking MBP immunostaining surrounding axonal NFL (red). **(C,D)** Immunostaining of differentiated oligodendrocyte progenitor cell (OPC) cultures derived from P15 *Tsc1^Flox^* **(C,C’,C”,C”’)** and *Tsc1^cKO^* **(D,D’,D”,D”’)** mice, with antibodies again MBP (green) and Cnpase (red). DAPI was used as the labeling for nuclei. **(E,F)** Western blot analysis of MBP, myelin-associated glycoprotein (MAG) and PDGFRa proteins in SC **(E)** and MBP, MAG and P0 in sciatic nerves **(F)** from P15 *Tsc1^Flox^* and *Tsc1^cKO^* mice. β-actin was used as loading control. **(G)** Quantification of immunoblots shown in **(E,F)**, *p < 0.05, or **p < 0.01. **(H)** RT-qPCR analysis of key differentiation genes in SCs from P15 *Tsc1^Flox^* (green) and *Tsc1^cKO^* (red) mice. Comparisons between genotypes were performed by Student *t*-test. *n* = 5, *p < 0.5. Scale bar: **(A–E)** = 2 μm.

Various TSC mouse models have been generated which generally show larger brain sizes often associated with myelin defects and hypomyelination, including loss of *Tsc1* in neurons (Meikle et al., 2007; Goto et al., 2011), and glial cells (Carson et al., 2015; Beirowski et al., 2017). We sought to examine the myelin deficits in greater detail and how loss of Tsc1 affected myelin ensheathment of axons using immunostaining of sciatic nerves from P15 *Tsc1^cKO^* mutants and control littermates. As shown in Figure 2A, in control nerves all axons displayed normal myelination, as revealed by immunostaining against the MBP (green) surrounding the axons immunostained against Neurofilaments (NFL; MBP with NFL, red; Figure 2A’). In *Tsc1^cKO^* mutant sciatic nerves, many axons irrespective of their size showed little or no staining for MBP and remained completely un-myelinated (MBP with NFL; Figures 2B,B’, arrows) indicating that loss of *Tsc1* in Schwann cells affects axonal myelination. We next tested for the expression MBP in *in vitro* OPC cultures from *Tsc1^cKO^* mutants and control littermates. In control OPCs, MBP staining (Figures 2C, 2C, C”’, green) mostly overlapped with Cnpase (CNP; Figures 2C’, 2C’, C”’ CNP, red). The nuclear stain DAPI served as the marker to locate cell nuclei (Figures 2C”,D”). In OPCs derived from *Tsc1^cKO^* mutants, the MBP staining was remarkably reduced (Figures 2D,D”’, green), as was the staining for Cnpase (Figures 2D’,D”’, red). These data show that loss of Tsc1 in myelinating glia affects the level of myelin-related proteins. To further establish how the level of myelination was affected, we carried out immunoblot analysis of SCs (Figure 2E) and sciatic nerves (Figure 2F) from *Tsc1^cKO^* mutants and control littermates. As shown in Figure 2E, both the 23 kDa and the 18 kDa isoforms of MBP were significantly reduced in *Tsc1^cKO^* mutants compared to controls in the SCs (36% reduction) and sciatic nerves (43% reduction; data quantified in Figure 2G). Myelin-associated glycoprotein (MAG) expression in the myelin sheaths in both CNS (Figures 2E,G) and PNS (Figures 2F,G) was also found to be reduced in both the SCs (32% reduction) and the sciatic nerves (50% reduction) of mutant animals, compared to control littermates. The myelin protein zero (MPZ or P0) was also significantly reduced in the sciatic nerves of the mutant mice (45% reduction; Figures 2F,G) confirming the *Cnp-Cre*-dependent loss of Tsc1 in the Schwann cells and its impact on myelin-related proteins in the PNS.

Next, we wanted to determine the stage of OPC differentiation in the SCs at which Tsc1 function may be critically required in myelinating glial cells. Immunoblot analysis of PDGFRa showed no significant changes in the protein levels between *Tsc1^cKO^* mutant SCs and control littermates (Figures 2E,G). As *Cnp* gene is only turned on in mature myelinating oligodendrocytes, these observations are consistent with previous reports of various* Cnp-Cre* mediated conditional knockout mouse model (Howng et al., 2010; Mei et al., 2013) in which PDGFRa^+^ OPCs remained unchanged in mutant mice compared to controls. We also prepared total RNA from *Tsc1^cKO^* mutant and control SCs and carried out qRT-PCR (Figure 2H). We used established OPC differentiation stage-specific markers to determine which markers are affected (Robinson et al., 2014). As show in Figure 2H, the progenitor and differentiating stage markers were not affected in *Tsc1^cKO^* mutant SCs, only the myelinating stage markers *UDP galactosyltransferase 8a* (*Ugt8a*) and *MBP* were significantly downregulated. These data indicate that early differentiation of OPCs in *Tsc1^cKO^* mutant SCs occurs normally and that only the myelinating phase is severely affected leading to hypo-myelination.

### Ultrastructural Analysis Reveals Severely Hypomyelinated Axons and Disorganized Peripheral Nerve Remak Bundles

**Figure 3:**
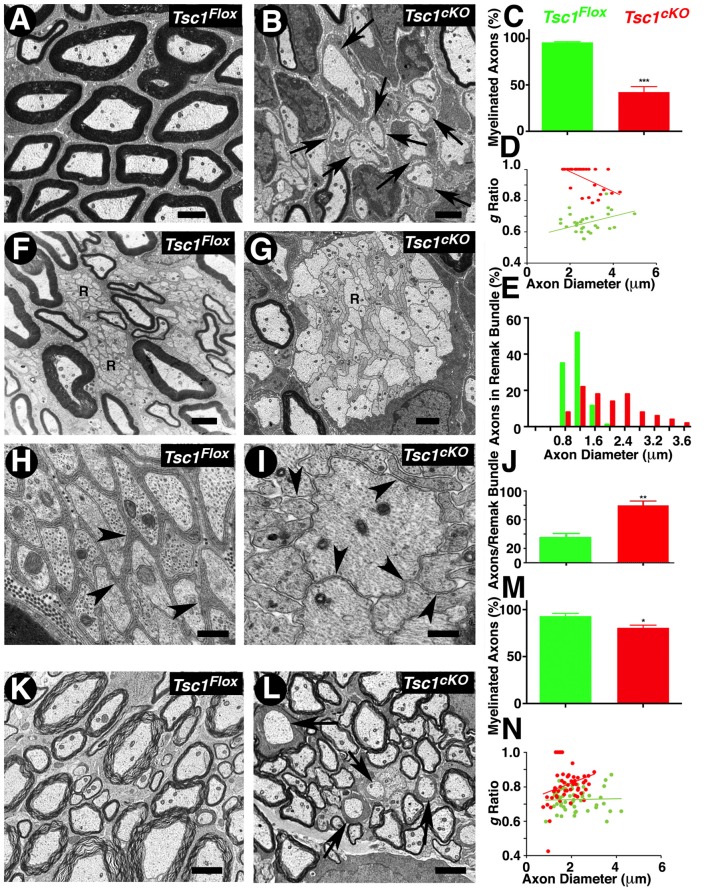
Ultrastructural analysis reveals severely hypomyelinated axons and disorganized peripheral nerve remak bundles. **(A–D)** Transmission electron microscopy (TEM) of cross sections from the sciatic nerve of P15 *Tsc1^Flox^* **(A)** and *Tsc1^cKO^* **(B)** mice. Morphometric analysis of myelinated axons in sciatic nerves, showing the percentage of myelinated axons **(C)** and the g-ratio values **(D)**. **(F–J)** TEM of cross sections, focusing on Remak bundles from sciatic nerve of P15 *Tsc1^Flox^* **(F,H)** and *Tsc1^cKO^* **(G,I)** mice. Axon diameter distribution and the number of axons per Remak bundle are shown in **(E,J)**, respectively. **(K–N)** TEM of cross sections from SC of P15 *Tsc1^Flox^* **(K)** and *Tsc1^cKO^* **(L)** mice. Morphometric analysis of myelinated axons in the SC showing the percentage of myelinated axons **(M)** and the g-ratio values **(N)**. Scale bars: **(A,B,K,L)** = 2 μm; **(F,G)** = 1 μm; **(H,I)** = 0.4 μm, *p < 0.05, **p < 0.01 or ***p < 0.001.

To further establish the extent of myelination and any ultrastructural abnormalities, which are not visible at the light microscopic level, we carried out TEM of P15 sciatic nerves and SCs from *Tsc1^cKO^* mutants and control littermates (Figure 3). The sciatic nerves from *Tsc1^cKO^* mutants showed a severe reduction of myelinated axons and a high number of barely myelinated or unmyelinated axons (Figure 3B, arrows), whereas the sciatic nerves from control littermates showed all properly myelinated axons (Figure 3A). Quantification of the hypomyelination phenotype showed that 60% of the axons were unmyelinated or thinly myelinated and never reached the level of myelination that was observed in controls (Figure 3C; compare Figure 3B with 3A). We also estimated the myelinated axon *g*-ratio (axon diameter/nerve fiber diameter) in *Tsc1^cKO^* mutant and control nerves. As shown in Figure 3D, the *g*-ratio was significantly higher in *Tsc1^cKO^* mutants than in control littermates, further demonstrating that the thickness of myelin sheaths was significantly reduced. Furthermore the mutant axons showed a trend towards increased diameter when compared to control, as quantified in Figure 3E. These data show that loss of Tsc1 in the Schwann cells severely affected myelination of the peripheral nerves.

Additional ultrastructural phenotypes observed in the peripheral nerves were the disorganization of Remak bundles, which are composed of a number of axons that remain unmyelinated and are surrounded by a thin membrane sheath from non-myelinating Schwann cells. In control littermates, unmyelinated axons in Remak bundles were well-organized with a regular size (<1.2 μm; Figure 3F, arrows); whereas in *Tsc1^cKO^* mutant, the axons in Remak bundles appeared to be abnormally shaped and disorganized, and remained unensheathed with a significant increase in the number of larger diameter axons (>2 μm). Some axons showed significantly large sizes and were also un-ensheathed (Figure 3I arrow, compare with Figure 3H). Moreover, in Remak bundles from *Tsc1^cKO^* mutants, the number of small diameter fibers significantly increased compared to the control littermates, indicating that loss of Tsc1 led to a failure of non-myelinating Schwann cells to ensheath unmyelinated axons quantified in Figure 3J). All the above changes in Remak bundles were observed at P15, prior to the completion of radial sorting by Schwann cells, suggesting that activated mTORC1 at the earlier developmental stages already had a significant impact on the radial sorting by Schwann cells. Surprisingly, *Raptor* mutants in which mTORC2 is deactivated (Norrmén et al., 2014) also revealed defects in radial sorting and enlarged immature Remak bundles before P14. Thus, mTORC1 and mTORC2 may have unique roles in radial sorting and proper Remak bundle formation. Additional pathologies observed in *Tsc1^cKO^* mutant nerve fibers were enlarged Schwann cells with their cytoplasms filled with accumulations of dense organelles appearing abnormal, such as rough endoplasmic reticulum (RER), mitochondria, or other unidentified cellular debris (data not shown). Similarly, in the CNS, the number of myelinated axons in the P15 SCs of *Tsc1^cKO^* mutants was also reduced by ~30% compared with control littermates (Figure 3L, arrows, compare with Figure 3K, arrows, quantified in Figure 3M). Estimation of *g*-ratio in the SC also showed an increase in the *g*-ratio of axons from *Tsc1^cKO^* mutants compared to control SCs (Figure 3N). Together, the ultrastructural analyses show that loss of Tsc1 affects the ensheathing properties of myelinating glial cells in both the PNS and CNS. These data further demonstrate the role of mTORC1 signaling in the proper organization of Remak bundles, thus highlighting the role of mTORC1 signaling in non-myelinating Schwann cells that has not been previously reported.

### Loss of Tsc1 in Myelinating Glia Leads to Disruption in Paranodal Domain Organization

**Figure 4:**
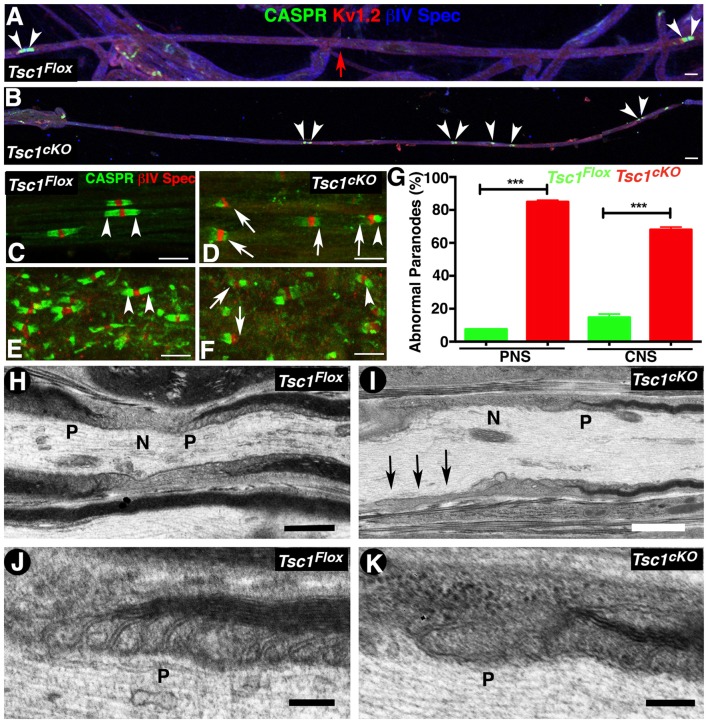
Loss of Tsc1 in myelinating glia leads to paranodal domain disorganization. **(A,B)** Immunostaining of sciatic nerve fibers from P15 *Tsc1^Flox^* **(A)** and *Tsc1^cKO^* **(B)** mice. Immunostaining with anti-Caspr paranodes (green, arrowheads), anti-Kv1.2 for juxtaparanodes (red), and anti-βIV Spec for the nodes (blue). Red arrow indicates the point of merger of two image panels showing the length of the internode in control sciatic nerve fibers. **(C–G)** Immunostaining of teased sciatic nerve fibers **(C,D)** and SC **(E,F)** from P15 *Tsc1^Flox^* **(C,E)** and *Tsc1^cKO^* mice **(D,F)**. Arrowheads indicate the healthy paranodes stained against Caspr (green); while arrows mark abnormal paranodes in *Tsc1^cKO^* mice. Quantification of the percentage of abnormal paranodes is shown in **(G)**, ***p < 0.001. Scale bars **(A–F)** = 6 μm. **(H–K)** TEM images of longitudinal sections of sciatic nerves from P15 *Tsc1^Flox^* **(H,J)** and *Tsc1^cKO^* mice **(I,K)**. N indicates the nodal area and P marks the paranodal regions. Black arrows indicate absence of the paranodal region in *Tsc1^cKO^* mice. Higher magnification images showing paranodal areas from *Tsc1^Flox^* **(J)** and *Tsc1^cKO^* **(K)** mice. Scale bars: **(H,I)** = 1 μm; **(J,K)** = 0.2 μm. *n* = 3 in each group.

The early onset of tremors in *Tsc1^cKO^* mutants at P10 and death around P15-P20 is very similar to that observed in Contactin-associated protein 1 (*Caspr1*; Bhat et al., 2001) or *Contactin* (Boyle et al., 2001), or *Nfasc^NF155^* mutants (Pillai et al., 2009) combined with reduced nerve conduction properties, which prompted us to analyze the myelinated nerve organization and whether axonal domains were compromised in myelinating glia-specific *Tsc1* mutants. We carried out immunostaining of teased myelinated fibers from P15 control and *Tsc1^cKO^* mutants using axonal domain specific markers for the node (Figures 4A,B, IV Spec, blue), the paranodes (Caspr, green) and the juxtaparanodes (K_v_1.2, red). Surprisingly, the paranodal axonal protein Caspr was either absent from the paranodes or present at some paranodes at much-reduced levels (Figure 4B, compare with control in Figure 4A). Interestingly, many myelinated axons displayed short internodes with small paranodal regions (Figure 4B, arrowheads, compare with Figure 4A, arrowheads). Quantification of the internodal length in the PNS myelinated axons as measured by the presence of the nodal/paranodal domains showed that loss of Tsc1 in the myelinating glia results in shorter internodes, about 30% reduction, compared to control myelinated axons. At a higher magnification, the PNS (Figure 4D, arrows) and the CNS (Figure 4F, arrows) myelinated axons showed missing or tiny paranodes as evidenced by Caspr immunostaining (CASPR, green) which was not observed in myelinated axons from control littermates in the PNS (Figure 4C, arrowheads) and CNS (Figure 4E, arrowheads). However, the distribution of nodal proteins, such as voltage-gated sodium channels (pan-Na_*V*_), βIV Spec and Ankyrin G (AnkG) seemed unaffected (data not shown), suggesting that the paranodal disorganization was occurring as a result of changes in the myelinating glial cells. Quantification of the paranodal abnormalities revealed that more than 80% of the *Tsc1^cKO^* mutant (Figure 4G) were lacking Caspr immunostaining at the paranodal regions indicating that loss of Tsc1 in the myelinating glia affected paranodal domain organization.

To better visualize the paranodal changes at the ultrastructural level, we performed TEM of the longitudinal sections of sciatic nerves from P15 *Tsc1^cKO^* mutants and control littermates. As expected, the paranodes (P) from control mice show the myelinating glial cell lateral loops spirally wrapped around the axon, and the glial membrane is closely apposed to the axolemma by septate-like junction structures (Figure 4H and at higher magnification Figure 4J). In *Tsc1^cKO^* mutants, the paranodal loops were either absent (Figure 4I, arrows, 4K, higher magnification) or were abnormally organized, as the transverse bands were much thinner and septate-like junctions were not properly formed or well defined. A majority of the paranodal regions examined (8 out of 10) lacked properly positioned paranodal structures and were often loose or completely disassembled. Together, these results show that ablation of *Tsc1* in myelinating glia affects the formation of the paranodal domain axo-glial junctions.

### Tsc1 Loss Leads to Downregulation of Neurofascin^NF155^ and Quaking RNA Binding Protein

**Figure 5:**
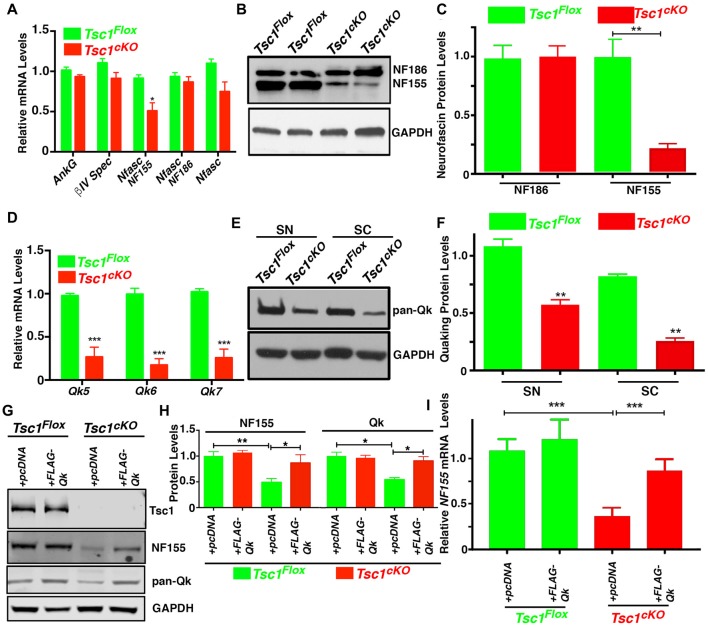
Loss of Tsc1 leads to downregulation of neurofascin^NF155^ and quaking (Qk) RNA binding protein. **(A)** RT-qPCR analysis for *AnkG, βIV Spec, Nfasc^NF155^, NFasc^NF186^* and total* Nfasc mRNA* from SCs from P15 *Tsc1^Flox^* (green) and *Tsc1^cKO^* mice (red). *n* = 3 in each group. **(B)** Western blot analysis of Nfasc^NF155^ and NFasc^NF186^ from P15 *Tsc1^Flox^* and *Tsc1^cKO^* SCs. *n* = 3 in each group. **(C)** Quantification of the levels of Nfasc^NF155^ and NFasc^NF186^. **(D)** RT-qPCR analysis for *Qk5*, *Qk6* and *Qk7* m*RNA* in SC from P15 *Tsc1^Flox^* (green) and *Tsc1^cKO^* mice (red). *n* = 3 in each group. **(E)** Western blot analysis of total Qk proteins in sciatic nerves (SN) and SCs from P15 *Tsc1^Flox^* and *Tsc1^cKO^* mice. *n* = 3 in each group. **(F)** Quantification data for band intensity of total Qk. *n* = 3 in each group. **(G,H)** Differentiated OPC cultures derived from P15 *Tsc1^Flox^* or *Tsc1^cKO^* mice were transiently transfected with *pcDNA* or *FLAG-Qk* plasmid. After 3 days of transfection, the total lysates were analyzed with antibodies for Tsc1, Nfasc^NF155^ and total Qk proteins **(G)**. Quantification data for band intensity of Nfasc^NF155^ and total Qk are shown in **(H)**. *n* = 3 in each group. **(I)** RT-qPCR analysis for Nfasc^NF155^ in differentiated OPC cells transfected with control or *FLAG-QK* plasmid. *n* = 3 in each group. Comparisons between genotypes or between different treatment within the same genotypes were performed by two-way ANOVA with Tukey Statistical post-test. *p < 0.05, **p < 0.01, ***p < 0.001.

Given the reduction in Caspr levels at the paranodal region in *Tsc1^cKO^* mutants, we wanted to determine whether these changes are as a result of changes in glial specific *Nfasc^NF155^* mRNA and protein expression, as Nfasc^NF155^ is essential for paranodal organization (Pillai et al., 2009). We carried out expression analysis of mRNAs encoding Nfasc^NF155^ and several other axonal nodal proteins from *Tsc1^cKO^* mutant and control SCs. As shown in Figure 5A, except for *Nfasc*^NF155^ mRNA which showed significant reduction, mRNAs for *AnkG*, *βIV Spec* and *Nfasc^NF186^* did not show any significant changes. Next, we used immunoblot analysis to quantitatively determine Nfasc^NF155^ and Nfasc^NF186^ protein level changes in *Tsc1^cKO^* mutants. As shown in Figures 5B,C, Nfasc^NF155^ protein levels were dramatically reduced in *Tsc1^cKO^* mutants whereas Nfasc^NF186^ levels were not altered (Figure 5C). These data indicate that both *Nfasc*^NF155^ mRNA and protein are specifically downregulated in *Tsc1*^cKO^ mutants.

Next, we sought to investigate the mechanism of how Nfasc^NF155^ was regulated upon the activation of mTORC1 signaling pathway as a result of loss of Tsc1 in myelinating glia. Both *Nfasc^NF155^* and *Nfasc^NF186^* are alternative splicing forms generated from the *Nfasc* locus (Pillai et al., 2009). As is well established, Nfasc^NF155^ is a glia-specific isoform (Tait et al., 2000; Charles et al., 2002; Pillai et al., 2009), whereas Nfasc^NF186^ is mainly produced as a neuronal isoform. Interestingly, a recent study reported that Quaking (Qk), an RNA binding protein is expressed in oligodendrocytes (Chen et al., 2007b) and Schwann cells (Larocque et al., 2009), and is involved in the regulation of the alternative splicing isoforms of the *Nfasc* gene, and particularly in the generation of *Nfasc*^NF155^, which is necessary for the formation of axo-glial junctions (Darbelli et al., 2016). We first determined whether there were any changes in the mRNA levels of the major *Qk* transcripts (*Qk5, Qk6* and* Qk7*) between *Tsc1^cKO^* mutants and controls. As shown in Figure 5D, mRNAs for all three *Qk* transcript were significantly downregulated in *Tsc1^cKO^* mutant SCs, when compared with control littermates. To determine whether Qk protein levels were affected, we carried out immunoblot analysis of both sciatic nerves and SCs of *Tsc1^cKO^* mutants and controls using a pan-Qk antibody. As shown in Figure 5E and quantified in Figure 5F, Qk total protein levels were significantly reduced in *Tsc1^cKO^* mutants compared to controls in both SCs and sciatic nerves (Figures 5E,F). These data suggest that loss of Tsc1 in myelinating glia causes downregulation of Qk protein, which in turn could affect the generation and/or stability of the *Nfasc*^NF155^ mRNA and consequently the levels of Nfasc^NF155^ protein, which specifically localized at the paranodal regions.

To further address a functional relationship between Qk and Nfasc^NF155^ in *Tsc1^cKO^* mutants, we prepared *in vitro* OPCs from both *Tsc1^cKO^* mutant and control mice, and stimulated the differentiation of OPC with T3 growth factor (Medina-Rodríguez et al., 2013). As shown in Figure 5G, the protein levels of Nfasc^NF155^ and Qk were significantly suppressed in differentiated OPCs from *Tsc1^cKO^* mutants, consistent with the changes that were observed *in vivo*. We then transiently introduced *pcDNA* control and* FLAG-Qk* plasmids (a gift from Thomas Tuschl, Addgene #19891) into differentiated OPCs from control and *Tsc1^cKO^* mutants. As shown in Figures 5G,H, expression of *Qk* in *Tsc1^cKO^* mutant OPCs restored the Qk protein levels and also restored expression of Nfasc^NF155^ protein as well as *Nfasc*^NF155^ mRNA (Figure 5I). These data demonstrate that loss of Tsc1 in oligodendrocytes and the consequent activation of mTORC1 pathway cause downregulation of Qk which then affects Nfasc^NF155^. Most importantly, expression of *Qk* which is downstream of *Tsc1* was able to restore Nfasc^NF155^ mRNA and the protein.

### Inhibition of mTORC1 by Rapamycin in the Absence of Tsc1 Rescues Paranodal Organization and Myelination

**Figure 6:**
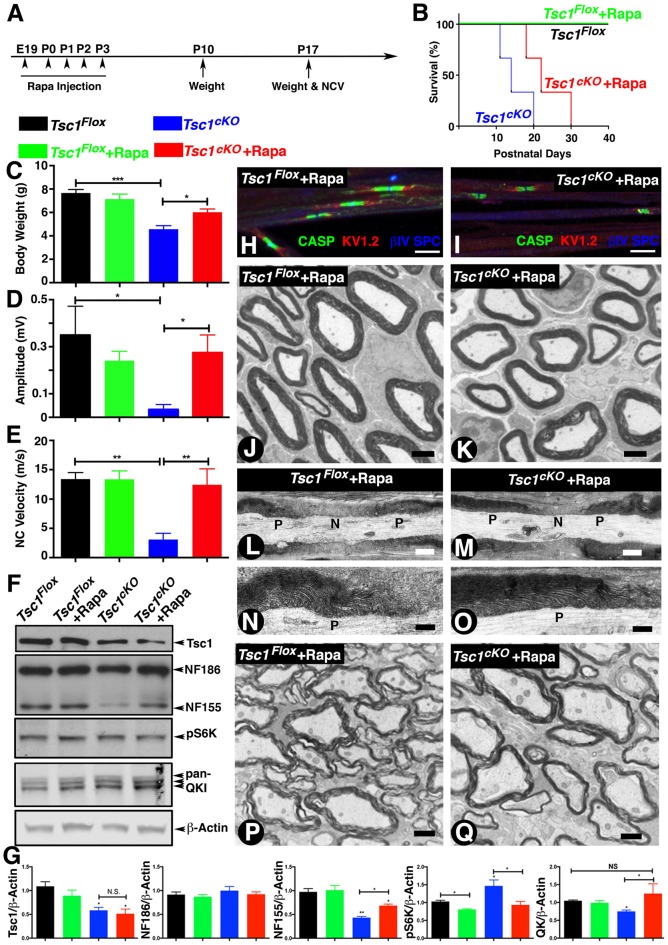
Inhibition of mTORC1 by Rapamycin in the absence of Tsc1 rescues nerve conduction, myelination and paranodal organization. **(A)**Timeline of Rapamycin administration (E19 to P3), weight measurements and end time points for experiments. *n* = 5 in each group. **(B)** Survival curve of *Tsc1^Flox^* and *Tsc1^cKO^* mice treated with Rapamycin (green and red), compared to mice without Rapamycin treatment (black and blue). *n* = 5 in each group. **(C–E)** Body weight measurement **(C)**, NCV **(D,E)** amplitude measurement of all four types of experimental group mice, *Tsc1^Flox^* and *Tsc1^cKO^*, *Tsc1^Flox^* and *Tsc1^cKO^* treated with Rapamycin. *n* = 5 in each group. **(F)** Western blot analysis of SC from all four types of experimental groups with antibodies against Tsc1, NF-CT, pS6K and pan-QK. **(G)** Quantification of immunoblots shown in **(F)**. β-Actin served as loading control. *n* = 3 in each group. **(H,I)** Immunostaining of teased SN fibers from control **(H)** and *Tsc1^cKO^* mutant mice **(I)** both receiving Rapamycin, with antibodies again Contactin-associated protein 1 (*Caspr1*; CASP, green), K_v_1.2 potassium channel (red) and nodal βIV Spectrin (blue). **(J,K)** TEM images of cross sections from sciatic nerve of P15 *Tsc1^Flox^* **(J)** and *Tsc1^cKO^* **(K)** mice, both receiving Rapamycin. **(L–O)** TEM images of longitudinal sections of sciatic nerves from P15 control **(L,N)** and *Tsc1^cKO^* Rapamycin-treated mice **(M,O)**. *n* = 3 in each group. **(P,Q)** TEM image of cross sections from SCs of P15 *Tsc1^Flox^* **(P)** and *Tsc1^cKO^* **(Q)** Rapamycin-treated mice. *n* = 3 in each group. Scale bars: **(G,H)** = 6 μm; **(I,J)** = 2 μm; **(K,L,O,P)** = 1 μm; **(M,N)** = 0.4 μm, *p < 0.05, **p < 0.01 or ***p < 0.001.

It is now well established that loss of Tsc1 in various cell types leads to constitutive activation of mTORC1 and that inhibition of mTORC1 by pharmacological treatments using Rapamycin blocks this signaling pathway (Kennedy and Lamming, 2016; Saxton and Sabatini, 2017a). We sought to determine whether various neuronal pathologies observed in *Tsc1^cKO^* mutants could be reversed by inhibiting mTORC1 activity by its well-known inhibitor, Rapamycin. Previous reports have shown that Rapamycin treatment is robust and prenatal injection in most of cases can significantly prolong lifespan of *Tsc* mutant mice (Li et al., 2014). As the myelinating glia-specific *Tsc1^cKO^* mutants rapidly develop severe neuronal defect as soon as P7 to P10 days and die at around P15, we designed a Rapamycin injection scheme as outlined in Figure 6A. We injected Rapamycin to *Tsc1^cKO^* mutant pregnant females daily at E19 and P0 to P3 (Figure 6A). The offspring were receiving Rapamycin only through lactation. Interestingly, this Rapamycin delivery scheme was enough to prolong the median lifespan of *Tsc1^cKO^* mutants from 15 days to 24 days (Figure 6B). At P15, the *Tsc1^cKO^* mutants receiving Rapamycin were indistinguishable from their control littermates by body weight (Figure 6C), and hind-limb paresis and tremor, and were almost not noticeable as mutant mice among all littermates, unlike the ones without Rapamycin (see Figure 1B). The *Tsc1^cKO^* mutants receiving Rapamycin had significantly improved mobility. Next, we carried out *in vivo* electrophysiological recordings from the sciatic nerves of *Tsc1^cKO^* mutants receiving Rapamycin to determine whether the nerve conduction properties were restored. As shown in Figures 6D,E, *Tsc1^cKO^* mutants without Rapamycin showed significantly reduced amplitude as noticed before; however, treatment with Rapamycin restored the amplitude to control levels. Similarly, the NCV was also restored upon Rapamycin treatment of *Tsc1^cKO^* mutants (Figure 6E). These data indicate that Rapamycin treatment of *Tsc1^cKO^* mutants, which lack Tsc1 in myelinating glia, is able to restore many behavioral deficits and nerve conduction properties in *Tsc1^cKO^* mutants.

Next, we carried out biochemical analysis to determine whether Rapamycin treatment was able to restore the protein levels of Nfasc^NF155^, Qk and pS6K to control levels. As shown in Figures 6F,G, the protein levels of Nfasc^NF155^ and Qk in *Tsc1^cKO^* mutants with Rapamycin were indistinguishable from those observed in control littermates (Figure 6F, quantified in Figure 6G). Furthermore, the inhibition of mTORC1 signaling in the* Tsc1^cKO^* mutants was also evidenced by the presence of comparable levels of phospho-S6 kinase (pS6K) levels across genotypes (Figure 6F, quantified in 6G). Consistently, the levels of Nfasc^NF186^ remained unaffected (Figure 6F, quantified in 6G). These data demonstrate that inhibition of mTORC1 by Rapamycin in the absence of *Tsc1* is able to rescue many deficits that occur as a result of activation of mTORC1 signaling pathway.

We then asked whether the paranodal structural changes and hypomyelination could be similarly rescued in *Tsc1^cKO^* mutants receiving Rapamycin. As shown in Figure 6I, Rapamycin treatment was able to restore paranodal staining of *Tsc1^cKO^* mutants and these paranodes were indistinguishable from those seen in control littermates (Figure 6H). Ultrastructural analysis of sciatic nerve cross sections (Figure 6K) and longitudinal sections (Figures 6M,O) from *Tsc1^cKO^* mutants receiving Rapamycin also showed a substantial improvement in the level of myelination of peripheral axons (compare Figure 6K with 6J). The paranodal structures at the paranodal regions were also much less disrupted and the morphology of these paranodes was similar to controls (Figures 6M,O, compare with Figures 6L,N). In addition, treatment with Rapamycin also restored myelination of the CNS myelinated axons in *Tsc1^cKO^* mutants (Figure 6Q, compare with 6P). Control littermates receiving Rapamycin did not show any significant changes in the body weight, NCV and amplitudes. Similarly, electron microscopic analysis did not show any significant changes in myelin thickness and the overall axonal domain structures including nodes and paranodes (*data no shown*). Together, these *in vivo* studies indicate that myelinating glia are susceptible to loss of Tsc1 leading to defective myelination and neurological disabilities, and that inhibition of mTORC1 by Rapamycin provides substantial benefits to overcome unwanted activation of mTORC1 signaling as a result of loss of Tsc1.

## Discussion

mTORC1 signal transduction pathway regulates a number of cellular processes that play a central role in cell growth and proliferation. In the current study, we have addressed the role of *Tsc1* in oligodendrocytes and Schwann cells. Animals that lack *Tsc1* in these cells showed severe behavioral and motor deficits and died around postnatal day 15 underscoring the importance of Tsc1 in animal survival. These animals also displayed hypomyelination and disorganization of the paranodal regions where axonal and myelin membranes come in close apposition to form axo-glial septate junctions. Most importantly, loss of Tsc1 led to downregulation of Quaking, an RNA binding protein that stabilizes mRNAs including that of glial *Nfasc*^NF155^ which was also downregulated. The downregulation of Nfasc^NF155^ protein is perhaps the underlying cause of the paranodal disorganization. Of significant interest is the finding that administration of Rapamycin, an inhibitor of mTORC1, was able to significantly rescue mutant phenotypes offers an opportunity to explore management of TSC-related myelin pathologies.

### Loss of Tsc1, mTORC1 Activation and Regulation of Axonal Myelination

Many mouse models have been recently generated with ablation of *Tsc1/2* in various types of cells including neurons and glial cells (Goto et al., 2011; Ercan et al., 2017; Zeng et al., 2008; Carson et al., 2015; Jiang et al., 2016). Specific ablation of *Tsc1* and/or *Tsc2* has been done in myelinating glial cells using either *Olig1-* (Jiang et al., 2016)* or Olig2-Cre* (Carson et al., 2015) for oligodendrocytes or *P0-Cre* for Schwann cells (Beirowski et al., 2017). Despite the hypermyelination phenotype discovered in some of the Akt/PTEN conditional knockout mice (Goebbels et al., 2010, 2012; Snyder et al., 2012; Kearns et al., 2015; Figlia et al., 2018), all TSC1/2 gene associated mutant animal models displayed hypomyelination with variable degrees of postnatal survival. It is clear that myelin-producing cells require *Tsc1/2* to maintain a fine balance of mTORC1 signaling and thus allow proper myelination. mTORC1 activation leads to downregulation of key myelin-related genes like *MBP* and *Ugt8a* which are required at late stages of myelination. Loss of Tsc1 using early *Olig1*- or *Olig2-Cre* also results in hypomyelination suggesting that mTORC1 activation is more detrimental towards the late phases of differentiation when myelination begins. Even though the activation of mTOR has been linked to upregulation of global protein synthesis, the down-regulation of major myelin proteins, such as MBP and P0, indicates the alternative regulation pathway responsible for mTOR up-regulation. It will be of significant interest to explore how mTORC1-dependent coordination of protein synthesis and degradation integrates with the necessary spatial and temporal control of key myelin protein translation. A detailed analysis of our current understanding of conditional genetic modified TSC mouse models and impact on myelination has been recently reported (Figlia et al., 2018).

Gene expression profiling of myelin-forming glial cells *Tsc1* mutants have uncovered a large number of genes that are up and downregulated (Jiang et al., 2016). While many transcription factors (Wang et al., 2014) play a critical role during myelination in myelinating glia to drive expression of myelin-related proteins, this must be coordinated with changes that also occur in glial cytoskeleton to allow growth of the cell membrane leading to axonal ensheathment and myelination. The hypomyelination phenotype in *Tsc1* mutants suggests an apparent paradox as mTORC1 signaling is typically considered to be a positive regulator of cell growth and differentiation (Saxton and Sabatini, 2017b). Although the hallmark of mTORC1 signaling activation in organogenesis is to accelerate cell growth, the present study, and many others (Lebrun-Julien et al., 2014; Carson et al., 2015; Jiang et al., 2016; Beirowski et al., 2017) have consistently shown that activation of mTORC1 signaling results in dysmyelination, in either OLs or SCs. Loss of Tsc1 using *Olig1*-cre showed increased apoptosis in OLs, which contributed to increased ER stress (Jiang et al., 2016); while deleting *Tsc2* by *Olig2*-Cre led to a fate shift from OL to astrocyte lineages (Carson et al., 2015). These above observations point to a critical time window in which neural development need to be finely coordinated with cellular functions especially when heterotypic cellular interactions between neurons and glia will depend on a fine balance of positive and negative regulation to achieve axonal myelination.

Another important phenotypic manifestation, as a result of loss of Tsc1, is the malformation and disorganization of the peripheral nerve Remak bundles, which are axonal tracks formed of unmyelinated axons and ensheathed by non-myelinating Schwann cells (Corfas et al., 2004). That fact that non-myelinating Schwann cell ensheathment is also affected in *Tsc1* mutants suggests that it is the ensheathment process in non-myelinating Schwann cells that requires axon sorting and ensheathment of axons to form Remak bundles. mTORC1 signaling has been shown to control neuropathic pain by negatively regulating low-density lipoprotein receptor-related protein (LRP1) Src homology 2/α-collagen (SchcA) complex in Schwann cells (Woldt et al., 2011). In *Lrp1* mutants, abnormal Remak bundles were observed with increase in the number and size of axons (Orita et al., 2013). Based on the ultrastructural observations, we did not see well defined glial membranes around the axons and often the entire bundle was disorganized with larger number of enlarged axons seen in the middle of the bundle. These phenotypes suggest that mTORC1 activation from loss of Tsc1 may have additional consequences on cellular functions that have not been observed previously.

In our current studies, *Cnp-Cre* mediated genetic ablation of *Tsc1* gene allowed the loss of Tsc1 in both oligodendrocytes (CNS) and Schwann cells (PNS). The *Cnp* locus has been well characterized and is expressed both in CNS, at the early postmitotic or mature myelinating oligodendrocytes (Trapp et al., 1988; Jiang et al., 2016), and in the PNS (Grigoryan et al., 2013). A recent study by Deng et al. (2014) showed that Cnp-mEGFP mice expressed GFP in the CNS, and in the sympathetic ganglia and DRG. Both in our current studies and previously (Pillai et al., 2009), we have demonstrated *Cnp-Cre* mediated recombination in both CNS and PNS. However, to separately analyze the impact of loss of Tsc1 in the Schwann cells in the peripheral nervous system, *P0-Cre* could be utilized, which may also allow better survival of eth mice to determine the long-term effects of Tsc1 loss in the PNS.

### mTORC1-Dependent Gene Regulation of Glial *Quaking* and *Neurofascin*

Under normal conditions when mTORC1 signaling is tightly controlled, myelinating glia serve the purpose of axonal myelination. From the expression analysis of various proteins it became evident that Qk, an RNA binding protein, transcripts and the protein were dramatically downregulated. Qk has been shown to be highly expressed in myelinating glia and proposed to directly regulate post-transcriptional homeostasis of key transcripts that are required for OPC differentiation and myelination (Chen et al., 2007b). Loss of Qk has also been shown to cause severe hypomyelination (Zhao et al., 2006). Of relevance to our observations, recent proteomic studies identified Qk as the potential downstream target for mTORC1 signaling (Tyler et al., 2011); however, a detailed mechanism of such regulation remains to be elucidated. During normal OPC differentiation, Qk levels gradually increase, while mTORC1 signaling is down-regulated allowing differentiation of myelinating glia to achieve myelination (Beirowski et al., 2017). It is thus conceivable that Qk, as an RNA binding protein, may play a major role in the splicing and/or stability of many glial transcripts that are required for differentiation and myelination, and thus may serve as a key factor that can regulate a large number of transcripts and their protein products. The finding that *Nfasc^NF155^* mRNA and protein were also downregulated and that re-expression of *Qk* in OPCs was able to restore both the mRNA and protein levels of Nfasc^NF155^ demonstrates that Qk controls *Nfasc*^NF155^ mRNA stability during differentiation, and that Qk may regulate other genes that are essential for myelination. Our results are consistent with Jiang et al. (2016); RNA-seq dataset: GSE47893) who have reported down-regulation of *Qk* and *Nfasc* mRNAs from optic nerves of *Olig1-Cre;Tsc1^Flox^* mutants. Our observations are also supported by a recent elegant study that showed a direct functional relationship between Qk and *Nfasc*^NF155^ isoform-specific transcripts in myelinating glial cells (Darbelli et al., 2016). The activation of mTOR signaling has been shown to positively regulate global protein synthesis by increasing eIF4F formation during cap-dependent translation (Zhang et al., 2014). However, the activation of mTOR can also promote protein degradation through multiple mechanisms, including upregulating catabolic signaling events or global ubiquitylation (Tang et al., 2014). In addition, mTOR signaling pathway has recently been shown to regulate microRNA biogenesis, and in turn regulates the transcript expression of target genes, providing a mechanism through which complex cellular functions can be coordinated (Bartel, 2009; Ogórek et al., 2018). The regulatory mechanisms of how mTOR signaling pathway regulates *Qk* gene by at the translational level need to be fully elucidated. Clearly the mRNA and protein levels of Qk are decreased in *Tsc1^cKO^* mice suggested a complex regulatory network in mTOR activated glial cell gene expression.

Additionally MBP has been known as the key target for Qk through nuclear export and mRNA stabilization (Larocque et al., 2002; Chen et al., 2007b). Thus down-regulation of Qk by activation of mTORC1 signaling leads to the reduced levels of both MBP mRNA and protein in *Tsc1* mutants. Stage-specific mRNA analysis of critical transcriptional factors revealed that *Cnp-Cre* mediated *Tsc1* deletion had no significant effects on early differentiating OLs; however, the myelinating stage OLs became severely dysfunctional highlighting a role for Tsc1 in differentiation. Together our studies uncover a link between mTORC1 signaling and regulation of Qk, a master stabilizer of RNA transcripts, during glial differentiation.

### mTORC1 Signaling and Glial/Axon Interactions and Paranodal Organization

Our findings that *Nfasc^NF155^* mRNA and protein are downregulated as a result of Qk downregulation indicate that myelination and axon-glial interactions are finely coordinated. While on one hand myelination is taking place, on the other axons and glia engage in a bidirectional interactions to coordinate the organization of axonal domains needed for saltatory conduction (Buttermore et al., 2013). Thus, when *Nfasc^NF155^* is downregulated by mTORC1 activation, paranodal domains fail to form properly as Nfasc^NF155^ is critically required for the paranodal domain organization (Pillai et al., 2009) along with axonal Caspr (Bhat et al., 2001). The paranodes serve as a membrane barrier that protects nodes and excludes juxtaparanodal components from invading into the nodal areas (Thaxton et al., 2011). The localization of Caspr at the paranodes depends on the presence of Nfasc^NF155^ proteins (Pillai et al., 2009), thus downregulation of Nfasc^NF155^ affects paranodal organization leading to changes in nerve conduction properties, as is observed in other mutants (Saifetiarova et al., 2017a,b), similar to *Tsc1^cKO^* mutant described here. Together our findings further underscore that mTORC1 activation has more global consequences on myelination and myelinated axon structure, which underlies deficits in nerve conduction properties and neurological phenotypes in *Tsc1* mutants.

### TSC-Associated Myelin-Related Pathologies

Defective myelination in TSC-associated neuropathologies underlies a number of pathologies and in addition disorganized axonal domains in TSC subjects will further lead to nerve damage and pathologies. Our phenotypic analyses of *Tsc1* mouse mutants combined with Rapamycin-administration and rescue analysis provide insights that some of the human phenotypes may be managed by tampering down the mTORC1 activation. Recently, several clinical trials were conducted to comprehensively examine the efficacy and safety of rapamycin-based therapy in TSC-associated diseases. Patients with lymphangioleiomyomatosis (LAM) or angiomyolipomas (AMLs), both of which are in association with TSC, show benefits from the use of rapamycin analogs (sirolimus or everolimus). However, once patients stopped taking rapamycin, their lung function began to deteriorate (McCormack et al., 2011), or the AML tumor recurred (Bissler et al., 2008, 2013) due to the transient binding/inhibitory effects of rapamycin towards mTOR complex. Our observations reveal that rapamycin treatment in the *Tsc1* mutant mice only prolonged the lifespan but did not prevent death of the mutant animal. While the effects of Rapamycin lasted as long as the administration was continued, the general health and quality of the mutant mice improved dramatically. Even though the effectiveness of rescue upon Rapamycin treatment in mutant animal is highly significant, the control littermates receiving rapamycin showed no significant changes compared to non-Rapamycin treated controls. These results are consistent with previous studies showing that Rapamycin treatment for 6 weeks or chronic treatment for 10 months in C57/BL6 wild type mice had very minimal effects on myelin thickness and number of myelinated axons (Narayanan et al., 2009; Nicks et al., 2014). In addition, genetic ablation of *mTOR* showed no major defects in myelination or myelin structural abnormalities (Sherman et al., 2012; Wahl et al., 2014). These results all highlight the remarkable role of Rapamycin in treating elevated mTORC1 induced pathologies, but not under normal physiological condition.

While Rapamycin is used in many human cancer patients, it remains to be seen whether TSC-associated myelin-related pathologies will show improvements in TSC subjects. Collectively, our studies provide a functional understanding of mTORC1 signaling regulating myelination and axonal domain organization and offer an avenue to manage myelin-related pathologies in TSC patients.

## Author Contributions

QS designed, performed experiments and wrote the article. JS and AMT performed the experiments. MB designed research and wrote the article.

## Conflict of Interest Statement

The authors declare that the research was conducted in the absence of any commercial or financial relationships that could be construed as a potential conflict of interest. The reviewer VM and handling Editor declared their shared affiliation.
